# Regulation of seed oil accumulation by lncRNAs in *Brassica napus*

**DOI:** 10.1186/s13068-022-02256-1

**Published:** 2023-02-10

**Authors:** Yuqing Li, Zengdong Tan, Chenghao Zeng, Mengying Xiao, Shengli Lin, Wei Yao, Qing Li, Liang Guo, Shaoping Lu

**Affiliations:** 1 National Key Laboratory of Crop Genetic Improvement, Huazhong Agricultural University, Wuhan, 430070 China; 2 Hubei Hongshan Laboratory, Wuhan, 430070 China; 3 Shenzhen Institute of Nutrition and Health, Huazhong Agricultural University, Wuhan, 430070 China; 4 Shenzhen Branch, Guangdong Laboratory for Lingnan Modern Agriculture, Agricultural Genomics Institute at Shenzhen, Genome Analysis Laboratory of the Ministry of Agriculture, Chinese Academy of Agricultural Sciences, Shenzhen, 518120 China

**Keywords:** LncRNA, Oil content, Lipid, Metabolite, Transgenic plants, *Brassica napus*

## Abstract

**Background:**

Studies have indicated that long non-coding RNAs (lncRNAs) play important regulatory roles in many biological processes. However, the regulation of seed oil biosynthesis by lncRNAs remains largely unknown.

**Results:**

We comprehensively identified and characterized the lncRNAs from seeds in three developing stages in two accessions of *Brassica napus* (*B. napus*), ZS11 (high oil content) and WH5557 (low oil content). Finally, 8094 expressed lncRNAs were identified. LncRNAs *MSTRG.22563* and *MSTRG.86004* were predicted to be related to seed oil accumulation. Experimental results show that the seed oil content is decreased by 3.1–3.9% in *MSTRG.22563* overexpression plants, while increased about 2% in *MSTRG.86004*, compared to WT. Further study showed that most genes related to lipid metabolism had much lower expression, and the content of some metabolites in the processes of respiration and TCA (tricarboxylic acid) cycle was reduced in *MSTRG.22563* transgenic seeds. The expression of genes involved in fatty acid synthesis and seed embryonic development (e.g., *LEC1*) was increased, but genes related to TAG assembly was decreased in *MSTRG.86004* transgenic seeds.

**Conclusion:**

Our results suggest that *MSTRG.22563* might impact seed oil content by affecting the respiration and TCA cycle, while *MSTRG.86004* plays a role in prolonging the seed developmental time to increase seed oil accumulation.

**Supplementary Information:**

The online version contains supplementary material available at 10.1186/s13068-022-02256-1.

## Background

Noncoding RNAs have been proved to play a critical role in regulating cell growth and development [1–3]. Genome-wide transcriptome sequencing studies have shown that over 90% of the eukaryotic genome can be transcribed, but only 1–2% of genes can code proteins, and a large proportion of genes in the genome are non-coding [4–6]. Based on the transcript length, the non-coding RNAs can be classified into short non-coding RNAs (< 200 nt) and long non-coding RNAs (lncRNAs, > 200 nt) [7, 8]. Based on the location of lncRNAs on the genome, they can be classified as intronic non-coding RNA, long intergenic non-coding RNA (lincRNA), sense lncRNA, and antisense lncRNA [9]. Previously studies have shown that many lncRNAs can affect the expression of their target mRNAs by *cis*—or *trans*-action, and then regulation of the gene function in cells [10]. It is believed that the lncRNAs drive their functions following three main principles: (1) lncRNA interacts with DNA, protein, or RNA molecules in cells as a functional biomolecule, (2) lncRNA sequences themselves contain the regulatory elements of other genes, thus influencing the gene expression by affecting the activity of lncRNA, and (3) influencing the genome during transcription and subsequently affecting the activity of gene [11].

Initially, lncRNAs were thought to be the transcriptional junk without any function. Since the discovery in the late 1980s that *H19* functions as an RNA in mammals, the role of lncRNAs has received more attention [12]. Over the past few decades, an increasing number of lncRNAs have been found to play key roles in maintaining the normal function of animal cells [13–15]. Recently, a large number of lncRNAs have also been identified in many plants, such as 6480, 2224, 14,749, 50,873, 20,163, 3181, 1910, and 5485 lncRNAs were predicted in Arabidopsis, rice, cotton, peanut, maize, rapeseed, *Camellia oleifera*, and tung tree, respectively [16–23]. Some lncRNAs in plants were functionally characterized to associate with plant growth, development, and response to abiotic and biotic stresses. For instance, two lncRNAs, *COOLAIR* and C*OLDAIR*, were reported to relate to the vernalization acting as a floral repressor in Arabidopsis [24]. Alternative Splicing Competitor RNA (*ASCO-*RNA) in Arabidopsis could competitively bind the nuclear speckle RNA-binding protein (NSR) and interfered with the alternative splicing of *NSR* downstream auxin-responsive genes, resulting in affecting the growth of lateral roots [25]. However, the function and action mechanism of most lncRNAs are still unknown [26].

Rapeseed (*B. napus*) is the third-largest oilseed crop in the world, accounting for about 13% of global oil production (USDA). Previous studies have well elucidated that the action mechanism of many transcriptional factors and genes are involved in seed oil biosynthesis [27–30]. Furthermore, studies revealed that some genes participated in the regulation process in oil accumulation [31, 32]. Recently, lncRNAs were also predicted to involve in regulating the seed oil biosynthesis. For example, in the developing seeds of *B. napus*, the expression patterns of 13 lncRNAs were significantly correlated with the expression patterns of 8 lipid-related genes, speculating these lncRNAs might have roles in oil synthesis [33]. However, there is no direct evidence that lncRNA takes part in the regulation of seed oil synthesis, and how the lncRNA impacts seed oil accumulation is still not clear.

In this study, we totally identified 12,973 lncRNAs in 3 different developing seed stages in 2 accessions *B. napus*, ZS11 (high oil content), and WH5557 (low oil content). Finally, 8094 expressed lncRNAs were remained after removing the alternative splicing. The analysis of lncRNAs–mRNAs co-expression and GO enrichment showed that cluster 2 and cluster 4 were involved in the processes of fatty acid biosynthesis and chloroplast stroma, respectively. According to the change trends of the expression levels of lncRNA and mRNA in different developing seed stages in two accessions, *MSTRG.22563* and *MSTRG.86004* from cluster 2 and cluster 4 were selected as the candidate lncRNAs to be investigated for the role in seed oil accumulation in *B. napus*. The results showed the oil content was decreased in *MSTRG.22563* overexpressed plant seeds but increased in *MSTRG.86004* overexpressed plant seeds. Moreover, the fatty acid composition and the expression of lipid mechanism related genes were changed in these two lncRNAs transferred seeds. Together, our results indicate that lncRNAs have roles in regulating oil accumulation and fatty acid composition in seeds of *B. napus*.

## Results

### Identification and characterization of the lncRNAs in developing seeds of *B. napus*

**Fig. 1:**
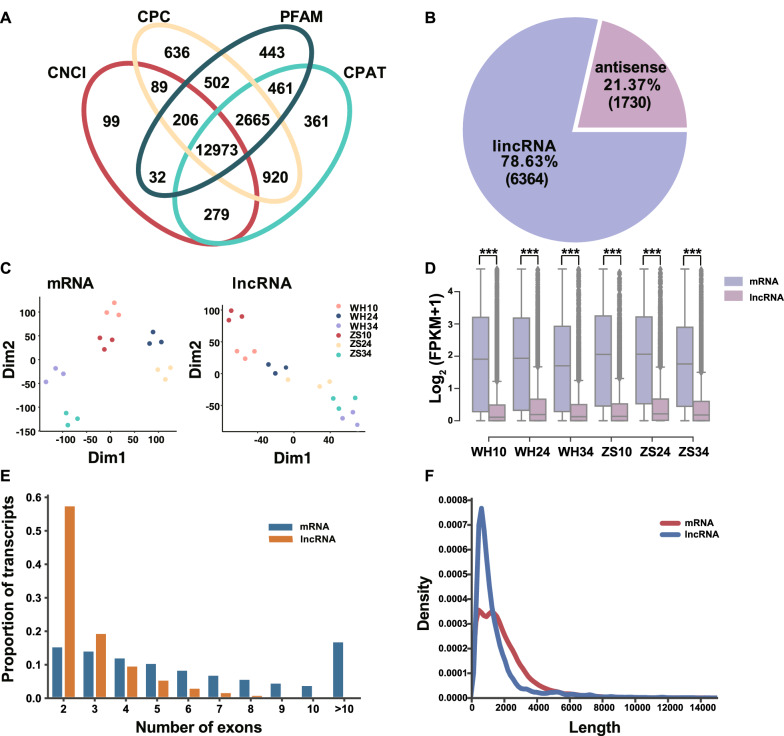
Identification of lncRNAs at 10 d, 24 d, and 34 DAF seeds of ***B. napu***s ZS11 (high oil content) and WH5557 (low oil content). A The Venn diagram shows the number of lncRNAs obtained by Coding–Non-Coding Index (CNCI) analysis, Coding Potential Calculator (CPC) analysis, PFAM protein structural domain analysis, Coding Potential Assessment Tool (CPAT). B Pie chart shows the different types of lncRNAs. **C** The analysis of t-SNE of mRNA-seq and lncRNA-seq in different developing seeds. Different color dots representation of different seed stages in two accessions. The same color dots representation of three replicates in each seed stage of the same accession. **D** Comparison of differentially expression levels of lncRNA and mRNA normalized using a formula of log_2_ (FPKM + 1). *** denote significance at *P* < 0.001 based on the student’s *t-*test. **E** The proportion of the different exon number of lncRNA and mRNA in transcripts. **F** The transcript length distribution of lncRNA and mRNA

ZS11 and WH5557 are two *B. napus* accessions with the high oil content and low oil content, respectively [34]. Three developing seed stages including 10, 24, and 34 DAF were used to do the transcript sequencing. Qualified RNA was sequenced in an Illumina instrument with an average data volume was 7.84 x. The workflow of lncRNAs and mRNAs analysis is shown in Additional file 1: Fig. S2. Subsequently, the transcripts judged as lncRNAs which existed in the four analysis methods including CNCI, CPC, CPAT, and PFAM. Totally, 12,973 lncRNAs were obtained (Fig. 1A, Additional file 2: Tables S1, S2, S3, S4, S5). After removal of the alternative splicing events, 8094 expressed lncRNAs were remained (Additional file 2: Table S6). Among them, there were 6364 lincRNAs (78.63%) and 1730 antisense lncRNAs (21.37%) (Fig. 1B, Additional file 2: Table S6). We also identified 24,160 expressed genes at different seed developmental stages in ZS11 and WH5557 (Additional file 2: Table S7). The t-SNE analysis showed that the mRNA and lncRNA at different seed development stages in different accessions could be separated clearly, and the repeatability of the three samples was great (Fig. 1C).

To further analyze the molecular characteristics of lncRNAs, we analyzed the information details of lncRNA and mRNA. Compared to mRNA, the expression levels of lncRNAs were much lower in all samples (Fig. 1D, Additional file 2: Table S7, Additional file 2: S8). The number of exons was fewer in most lncRNAs than that in mRNAs. The highest proportion of lncRNA contained two exons, followed was three exons, the counts of them were near 60 and 20% in total lncRNAs, respectively (Fig. 1E). However, the highest proportion of mRNA contained more than 10 exons, followed was two, three, four, and five, and their counts were 17.0, 15.5, 14.3, 12.2, and 10.6% in total mRNAs, respectively (Fig. 1E). In addition, the average length of lncRNAs was also significantly shorter than that of mRNAs (Fig. 1F).

**Fig. 2:**
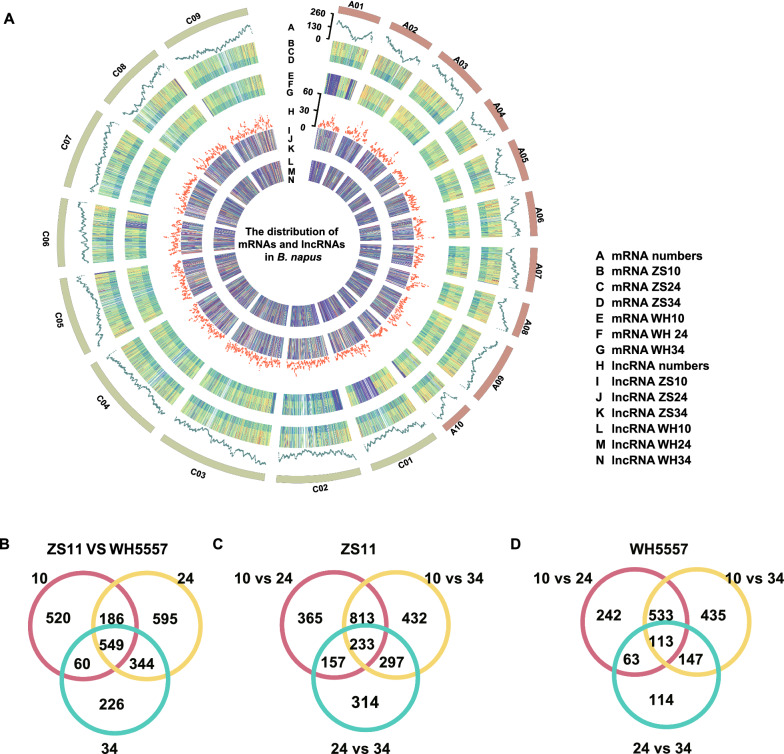
Comparisons of lncRNAs and protein-coding mRNAs in *B. napus*. **A** Distribution of the number and expression levels of lncRNAs and mRNAs on chromosomes. The scale representation of the number of lncRNAs and mRNAs on the chromosome. Different letters representation of the expression of lncRNA and mRNA in different seed stages in two accessions. **B** Venn diagram showing differentially expressed lncRNAs in different accessions in the same seed developmental stage. **C** and **D**, Venn diagram showing differentially expressed lncRNAs in the same accession among three different seed developmental stages

We also compared the distribution of expression levels and numbers of lncRNA and mRNA on chromosomes. The results showed that most lncRNA enrichment regions on the chromosomes were not consistent with that of mRNA enrichment (Fig. 2A). Furthermore, the expression levels of lncRNAs were observed significantly lower than that of mRNAs in the same location on the chromosomes at the same seed development stage of both ZS11 and WH5557, but in several regions like on C01, C02, C03 and A01 in WH5557, the expression of mRNAs was as low as lncRNAs (Fig. 2A). It seemed that there was no visual difference in the expression change trends of lncRNA at different seed development stages of ZS11 compared to WH5557 from the whole map, and the same trends also existed in mRNA except for several low expressed regions on C01, C02, C03 and A01 in WH5557 (Fig. 2A). We compared the expression levels of lncRNA among 10, 24, and 34 DAF seeds in the same accession, and the expression levels of lncRNA at the same seed development stage between ZS11 and WH5557. The differential transcript levels of lncRNA with |log_2_foldchange|≥ 1 and *q* < 0.05 were selected to draw a Venn diagram. There were 549 lncRNAs differentially expressed among 10, 24, and 34 DAF seeds of ZS11 and WH5557 (Fig. 2B), 233 lncRNAs in ZS11 (Fig. 2C), and 113 lncRNAs in WH5557 (Fig. 2D).

### Analysis of differentially expressed lncRNAs in different seeds’ developmental periods of two *B. napus* accessions

**Fig. 3:**
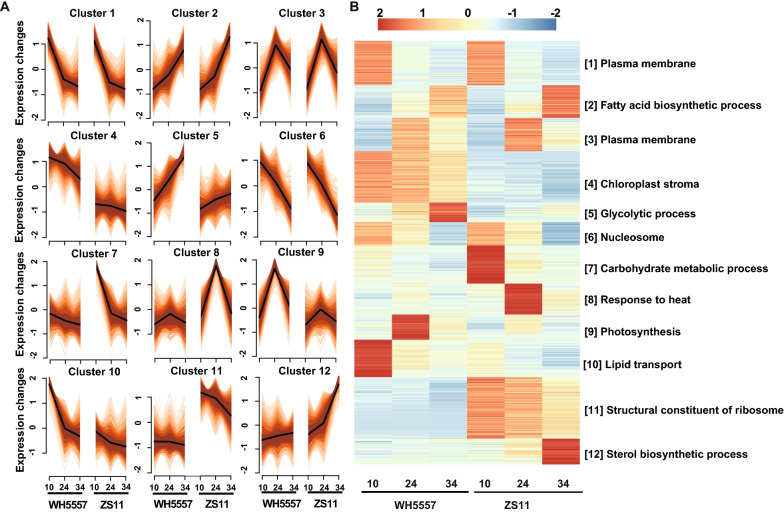
Dynamic changes of lncRNAs and mRNAs during seed development in *B. napus*. **A** Cluster analysis of all lncRNAs and mRNAs based on their expression level change patterns using Mfuzz. **B** Heat map analysis showing the differentially gene expressed internal different modules

To further clarify the interrelationship between lncRNA and mRNA, we used Mfuzz to classify the expression patterns of lncRNAs and mRNAs according to whether they had the same expression change trends at different seed development stages. Finally, 12 different expression patterns were clustered (Fig. 3A). Then, the expression levels in each cluster were extended using a heat map and performed with GO enrichment (Fig. 3B). The analysis results showed that the clusters from 1 to 12 were mainly enriched in the process of the plasma membrane, fatty acid biosynthesis, plasma membrane, chloroplast stroma, glycolysis, nucleosome, carbohydrate metabolism, response to heat, photosynthesis, lipid transport, structural constituent of ribosome, and sterol biosynthesis, respectively (Fig. 3B, Additional file 2: Table S9, S10, S11, S12, S13, S14, S15, S16, S17, S18, S19, S20). The top 10 GO terms of each cluster are displayed in Additional file 1: Fig. S3. The GO terms of cluster 7, 10, and 12 were less than 10, and GO graphs of them were plotted according to the FDR (False Discovery Rate) value from the smallest to the largest. The fatty acid biosynthesis main occurs in the plastid of plants, and the synthesized fatty acid is the resource for TAG assembly. Hence, cluster 2 and cluster 4 were selected to screen the candidate lncRNAs which perhaps impact seed oil accumulation.

### Prediction of the candidate lncRNAs related to oil accumulation

**Fig. 4:**
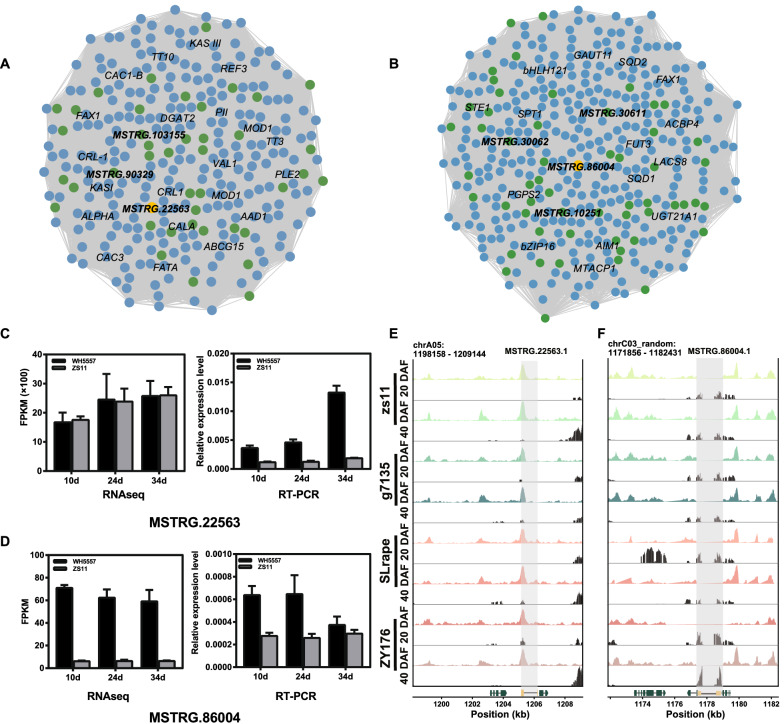
The co-expression analysis of lncRNAs and mRNAs and the selection of candidate lncRNAs. **A** Co-expression network analysis in cluster 2. Green circles represent lncRNA, blue circles represent mRNA, the yellow circle is *MSTRG.22563*. **B** Co-expression network analysis in cluster 4. Green circles represent lncRNA, blue circles represent mRNA, the yellow circle is *MSTRG.86004*. C and D, The expression levels of *MSTRG.22563* and *MSTRG.86004* from RNA-seq data and qRT-PCR data. Relative expression level in qRT-PCR was calculated by comparison to actin’s expression level. Values are means ± SD (*n* = 3). **E** and **F**, The distribution and expression patterns of *MSTRG.22563* and *MSTRG.86004* in 20 and 40 DAF seeds of four *B. napus* accessions in ATAC-seq analysis. The peaks representation of the expression of transcripts, color figures representation of the expression of lncRNAs in the region, black figures representation of the expression of mRNAs in the region. The structure colored with bottle-green represents genes, the structure colored with yellow represents lncRNAs. ATAC-seq, Assay for Transposase-Accessible Chromatin with high-throughput sequencing

LncRNAs–mRNAs co-expression networks with the value of the correlation > 0.9 in cluster 2 and > 0.98 in cluster 4 were drawn according to the change trends of the transcript’s expression during seed development, respectively (Fig. 4A, B). The results showed that 42 lncRNAs and 257 mRNAs in cluster 2 had highly correlation (Fig. 4A, Additional file 2: Table S21), and 57 lncRNAs and 352 mRNAs in cluster 4 had highly correlation (Fig. 4B, Additional file 2: Table S22). There were many genes related to the process of fatty acid or TAG synthesis or transcript factors (TFs) related to oil accumulation in the networks, such as *ketoacyl-ACP reductase I* (*KASI*), *ketoacyl-ACP reductase III* (*KASIII*), *diacylglycerol acyltransferase 2* (*DGAT2*), and *HIGH-LEVEL EXPRESSION OF SUGAR-INDUCIBLE GENE 2* (*VAL1*) in the co-expression network of cluster 2 (Fig. 4Aa), *LONG-CHAIN ACYL-COA SYNTHETASE 8* (*LACS8*), *STEROL 1* (*STE1*), *FATTY ACID EXPORT 1* (*FAX1*) and TFs such as *BASIC REGION/LEUCINE ZIPPER TRANSCRIPTION FACTOR 16* (*bZIP16*) and *basic helix-loop-helix121* (*bHLH121*) in the co-expression network of cluster 4 (Fig. 4B). The candidate lncRNAs which had the higher expression level both in two accessions or the larger differentially expressed in different accessions, and meanwhile the expression change trends were consistent in seed development in two accessions were screened. Hence, the *MSTRG.22563* in cluster 2 and the *MSTRG.86004* in cluster 4 were chosen as the candidate lncRNAs (Fig. 4C, D, Additional file 2: Table S21, S22). Further, the expression of *MSTRG.22563* and *MSTRG.86004* was verified by qRT-PCR (Fig. 4C, D). The expression pattern of *MSTRG.86004* in qRT-PCR was consistent with that in transcript sequencing. The expression of *MSTRG.22563* using qRT-PCR at the same seed development stage in ZS11 was significantly lower than that in WH5557, while that was no different in RNA-seq data (Fig. 4C). However, the expression change trends in three seed development of different accessions were consistent between RNA-seq data and qRT-PCR data (Fig. 4C, D). Furthermore, we analyzed the ATAC-seq (Assay for Transposase-Accessible Chromatin with high-throughput sequencing) data of these two candidate sequences at 20 DAF and 40 DAF seeds in four accessions of *B. napus*, ZS11, G7135, SLrape, and ZY176. The results showed that there was an open region in the first exon of *MSTRG.22563* (has two exons) in chromatin in all accessions (color figures), while there was almost no opened gene in this region (black figures) (Fig. 4E). However, the region of *MSTRG.86004* (has two exons) was not opened in chromatin in all accessions (color figures), but the gene at the two ends of this region obviously was opened (black figures) (Fig. 4F). These results suggest that these two lncRNAs might have different roles in seed development by different action mechanism.

### Overexpression of *MSTRG.86004* increased the oil content and changed the fatty acid composition of seeds

**Fig. 5:**
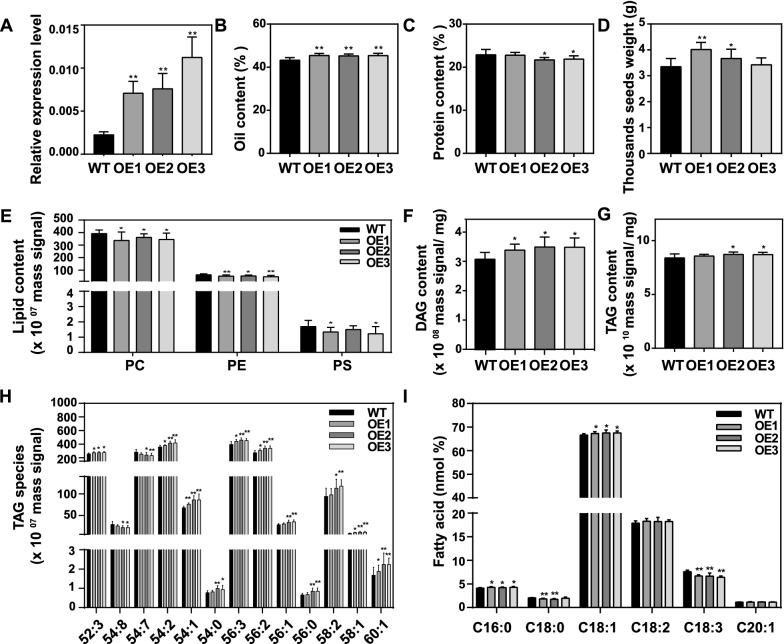
Overexpression of *MSTRG.86004* influenced seed oil content of *B. napus*. **A** *MSTRG.86004* transcript levels in 32 DAF seeds were detected by real-time PCR. Values are means ± SD (*n* = 3). **B** and **C**, Oil content and protein content of mature seeds were analyzed using NIR instruments. Values are means ± SD (*n* = 8–12). **D** The 1000 seed weight. Values are means ± SD (*n* = 8–12). **E**, **F** and **G**, The content of PC, PE, PS, DAG, and TAG were analyzed by LC–MS/MS. Values are means ± SD (*n* = 8–12). **H** The content of different TAG species. Values are means ± SD (*n* = 8–12). **I** The composition of fatty acid in mature seeds was analyzed by GC-FID. Values are means SD (*n* = 8–12). * and ** denote significance at *P* < 0.05 and *P* < 0.01, respectively, compared with WT based on the student’s *t*-test. PC, phosphatidylcholine; PE, phosphatidylethanolamine; PS, phosphatidylserine; DAG, diacylglycerol; TAG, triacylglycerol

The vectors containing *MSTRG.86004* and *MSTRG.22563* were transferred into the *Agrobacterium tumefaciens* (GV3101) which were used to infect the hypocotyls of *B. napus* cultivar Westar. Finally, three *MSTRG.86004* expressed lines (named OE1—OE3) and five *MSTRG.22563* expressed lines (named OE4—OE8) were obtained (Figs. 5, 8A). In the following study, the OE1, OE2, and OE3 were used to investigate the function of *MSTRG.86004* in seed oil synthesis, and the OE4, OE5, and OE7 were used to explore the function of *MSTRG.22563* in seed oil synthesis. The gene expression data showed that OE1, OE2, and OE3 seeds had higher *MSTRG.86004* expression levels than WT (Fig. 5A). After plants harvested, the traits such as plant height, inflorescence length, seed number per silique, and silique length were measured. There were no differences in plant height, inflorescence length, number of effective branches, silique length, seed number per silique between transgenic lines and WT (Additional file 2: Table S24). The oil content and protein content of transgenic seeds were investigated using a NIR machine. The results showed that the oil content of OE1, OE2, and OE3 was significantly increased by about 2% compared to WT (Fig. 5B), while the protein content of OE2 and OE3 was significantly lower than that of WT, but had no difference between OE1 and WT (Fig. 5C). Moreover, the 1000 seeds weight was increased by 19.9% and 9.5% in OE1 and OE2 compared to WT, respectively (Fig. 5D).

To understand which lipid changes in transgenic plant seeds, the lipid of mature seeds was analyzed by LC–MS/MS. The results displayed that the content of phosphatidylcholine (PC), phosphatidylethanolamine (PE), and phosphatidylserine (PS) in OE lines was significantly lower than that in WT, except there was no difference in PS between OE2 and WT (Fig. 5E). The content of lysoPE was also significantly lower in all OE seeds than that of WT, but there was no difference in lysoPC between OE seeds and WT (Additional file 1: Fig. S4). Neutral lipid includes DAG and TAG which are the main component of seed oil. The content of DAG in OE1, OE2, and OE3 was increased by 9.9, 13.5, and 13.2% than that of WT, respectively (Fig. 5F). The content of TAG was increased by 3.7 and 3.5% in OE2 and OE3 than that of WT, respectively, but no difference between OE1 and WT (Fig. 5G). The species of TAG-52:3, -54:2, -54:1, -56:3, -56:2, -58:1, and -60:1 were significantly higher in all OE lines than that in WT. The species of TAG-54:0, -56:1, -56:0, and -58:2 were significantly higher in OE2 and OE3 than that in WT. The species of TAG-54:8 and -54:7 were lower in OE2 and OE3 (Fig. 5G). To investigate which fatty acid changes in transgenic plant seeds, the methyl-esterified fatty acid was extracted and analyzed using a GC-FID machine. The result revealed that the content of C16:0 and C18:1 was significantly increased in OE1, OE2, and OE3 compared to WT. However, the C18:0 and C18:3 was significantly decreased in the OE lines, except C18:0 had no difference in OE3 compared to WT (Fig. 5I). Together above, the overexpression of lncRNA *MSTRG.86004* could increase the weight and oil content of seeds and change the fatty acid composition.

### The change of the expression levels of genes related to lipid metabolism in the *MSTRG.86004* overexpression seeds

**Fig. 6:**
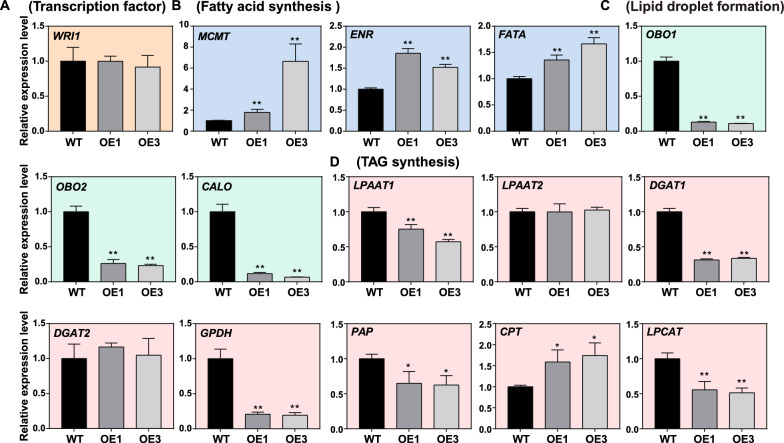
The expression levels of genes involved in lipid synthesis in the 32 DAF seeds of *MSTRG.86004* OE plants. **A** The expression levels of TF. *WRI*, *Wriknkled1*. **B** The expression levels of genes related to fatty acid synthesis. *MCMT*, *ENR*, and *FATA*. *MCMT*, *Malonyltransferase*; *ENR*, *Enoyl-ACP reductase*; *FATA*, *Acyl-ACP thioesterase A*. **C** The expression levels of genes participated in lipid droplet formation. *OBO1*, *Oil body oleosin 1*; *OBO2*, *Oil body oleosin 2*; *CALO*, *Caleosin*. **D** The expression levels of genes involved in TAG assembly. *LPAAT1*, *lysophosphatidic acid acyltransferase 1*; *LPAAT2*, *lysophosphatidic acid acyltransferase 2*; *DGAT1*, *diacylglycerol acyltransferase 1*; *DGAT2*, *diacylglycerol acyltransferase 2*; *GPDH*, *Glycerol-3-phosphate dehydrogenase*; *PAP*, *Phosphatidate phosphatase*; *CPT*, *Choline phosphotransferase*, *LPCAT*, *lysophosphatidylcholine acyltransferase*. Total RNA was extracted from 32 DAF seeds. Relative expression level was calculated by comparison to actin’s expression level. Values are means ± SD (*n* = 3). * and ** denote significance at *P* < 0.05 and *P* < 0.01, respectively, compared with WT based on the student’s *t*-test

To clarify how the *MSTRG.86004* affects the seed oil content of *B. napus*, the expression of some key genes related to the process of oil synthesis, such as TAG synthesis, lipid droplet formation, and fatty acid synthesis was examined in 32 DAF seeds. Genes such as *Wriknkled1* (*WRI1*), *lysophosphatidic acid acyltransferase* (*LPAAT*), *diacylglycerol acyltransferase* (*DGAT*), *glycerol-3-phosphate dehydrogenase* (*GPDH*), *phosphatidate phosphatase* (*PAP*), *lysophosphatidylcholine acyltransferase* (*LPCAT*), *oil body oleosin* (*OBO*), *Caleosin* (*CALO*), *Malonyltransferase* (*MCMT*), *enoyl-ACP reductase* (*ENR*), *CDP-choline: diacylglycerol cholinephosphotransferase* (*CPT*), and *Acyl-ACP thioesterase A* (*FATA*) participated in the processes of oil biosynthesis were tested by qRT-PCR. Compared to WT, there was no significant change in the expression level of *WRI1* in OE seeds (Fig. 6A). The expression levels of *MCMT*, *ENR*, and *FATA* genes related to fatty acid synthesis were significantly up-regulated in OE1 and OE3 compared to WT (Fig. 6B). However, the expression levels of *OBO1*, *OBO2*, *CALO*, *LPAAT1*, *DGAT1*, *GPDH*, *PAP*, and *LPCAT* were significantly suppressed in OE1 and OE3 (Fig. 6C, D). In addition, the expression levels of *LPAAT2* and *DGAT2* were no changes between OE and WT, but the *CPT* had higher expression in OE than WT (Fig. 6D). These results suggest that *MSTRG.86004* has a role in impacting the processes of fatty acid synthesis, TAG assembly and storage.

### The *MSTRG.86004* delayed seed development

**Fig. 7:**
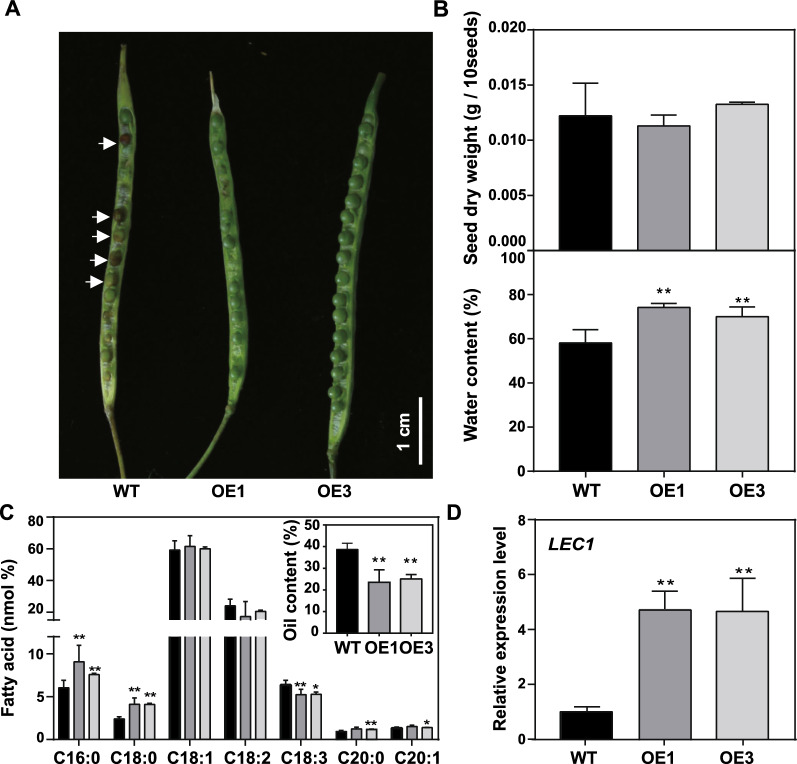
The comparison of 40 DAF seeds which pollinated at the same time in the similar position in *MSTRG.86004* OE and WT plants. **A** The phenotype of the 40 DAF seeds. **B** and **C**, The dry weight and water content of 40 DAF seeds. Values are means ± SD (*n* = 6–8). **D** and **E**, The oil content and fatty acid composition of 40 DAF seeds. Values are means ± SD (*n* = 6–8). **f** The expression levels of *LEC1* in 32 DAF seeds. Values are means ± SD (*n* = 3). * and ** denote significance at *P* < 0.05 and *P* < 0.01, respectively, compared with WT based on the student’s *t*-test. *LEC1*, *leafy cotyledon 1*

To explore how the *MSTRG.86004* increases the expression of gene related to fatty acid biosynthesis but decreases the expression of gene related to TAG assembly without disrupting the expression of *WRI1*, the 40 DAF seeds at the similar position of OE and WT plants were investigated. The seeds of OE were observed greener than that of WT (Fig. 7A, white arrows). The dry weight of the OE seeds was equal to that of WT, while the water content of OE seeds was heavier 12.2–16.5% than that of WT (Fig. 7B). In addition, the oil content of OE seeds was lower 13.0–15.2% compared to WT (Fig. 7C, top right corner), and the composition of C16:0, C18:0, and C18:3 was significantly changed (Fig. 7C). *Leafy cotyledon 1* (*LEC1*) and *LEC2* are the two marker genes related to seed development (Pelletier et al*.* 2017; Song et al*.* 2021). The expression of *WRI1*, *LEC1*, and *LEC2* in the whole seed development period of ZS11 was investigated (http://yanglab.hzau.edu.cn/BnTIR). The results displayed that the expression of *LEC1* and *LEC2* decreased seriously from 32 and 24 DAF, and their expression closed to zero at 54 DAF and 38 DAF, respectively. However, the expression of *WRI1* decreased a little from 34 DAF and then kept to the same levels till to 52 DAF (Additional file 1: Fig. S5A). The gene expression levels of *LEC1* and *LEC2* were tested in *MSTRG.86004* OE seeds by qRT-PCR, the results showed that the level of *LEC1* in both OE seeds was 5 folds than that in WT, while the expression of *LEC2* had no significant difference compared to WT (Fig. 7D, Additional file 1: Fig. S5B). Together, the results suggest that *MSTRG.86004* impacts oil accumulation by regulating seed development.

### Overexpression of *MSTRG.22563* decreased the oil content and changed the fatty acid composition of seeds

**Fig. 8:**
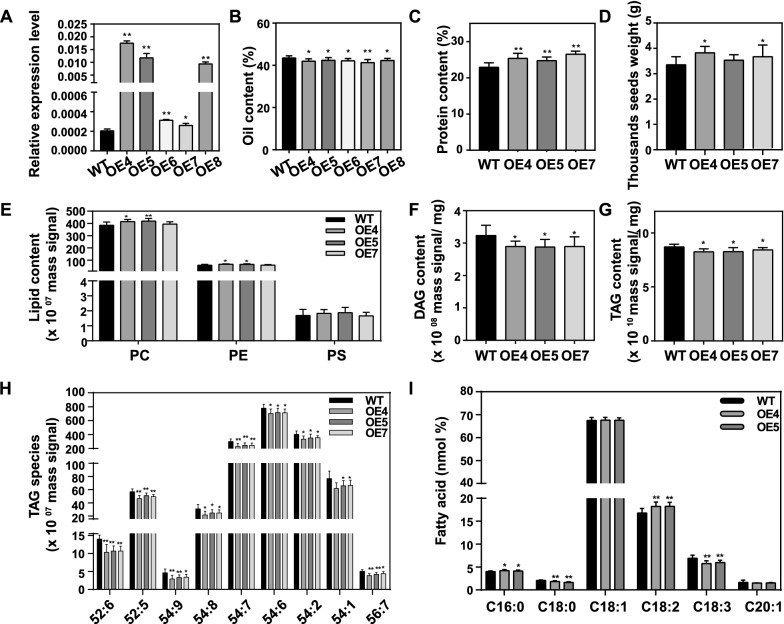
Overexpression of *MSTRG.22563* influenced seed oil content of *B. napus*. **A** *MSTRG.22563* transcript levels in 32 DAF seeds were detected by real-time PCR. Values are means ± SD (*n* = 3). **B** and **C**, Oil content and protein content of mature seeds were analyzed using NIR instruments. Values are means ± SD (*n* = 8–12). **D** The 1000 seed weight. Values are means ± SD (*n* = 8–12). **E**, **F** and **G**, The content of PC, PE, PS, DAG, and TAG were analyzed by LC–MS/MS. Values are means ± SD (*n* = 8–12). H The content of different TAG species. Values are means ± SD (*n* = 8–12). **I** The composition of fatty acid in mature seeds was analyzed by GC-FID. Values are means SD (*n* = 8–12). * and ** denote significance at *P* < 0.05 and *P* < 0.01, respectively, compared with WT based on the student’s *t*-test. PC, phosphatidylcholine; PE, phosphatidylethanolamine; PS, phosphatidylserine; DAG, diacylglycerol; TAG, triacylglycerol

The qRT-PCR result showed that all the transgenic seeds had a higher expression level of *MSTRG.22563* than WT (Fig. 8A). The oil content of OE4–OE8 mature seeds was decreased by 3.1–3.9% compared to WT (Fig. 8B). Then, OE4, OE5, and OE7 were used to do the subsequent analysis. The protein content of OE4, OE5, and OE7 was found to increase 10.7, 8.1, and 15.7% compared to WT (Fig. 8C). The 1000 seeds weight was also increased by 14.1 and 9.5% in OE4 and OE7 compared to WT, respectively, but had no difference between OE5 and WT (Fig. 8D). However, there were no differences in plant height, inflorescence length, number of effective branches, silique length, seed number per silique between transgenic lines and WT (Additional file 2: Table S24). In addition, the dry weight and water content of 40 DAF OE seeds were equal to that of WT (Additional file 1: Fig. S6).

LC–MS/MS was employed to measure the content of lipids in mature seeds. The content of PC and PE was significantly higher in OE4 and OE5 than that in WT, but no difference in OE7 (Fig. 8E). There was no difference in PS between all OE lines and WT (Fig. 8E). In addition, the content of lysoPC and lysoPE in OE seeds was higher than that in WT (Additional file 1: Fig. S7). The content of DAG and TAG was decreased about 10.0 and 3.5–4.9% in three OE lines than that of WT, respectively (Fig. 8F, G). The TAG species were compared among OE4, OE5, OE7, and WT. The species of TAG-52:5, -52:6, -54:6 ~ 54:9, -54:1, -54:2, and -56:7 in OE4, OE5, and OE7 were significantly lower than that in WT (Fig. 8H). The species of TAG-54:1 was lower in OE5 and OE7 compared to WT, but no difference between OE4 and WT (Fig. 8H). To understand which fatty acid changes in TAG, the fatty acid composition of OE4 and OE5 mature seeds was measured using the GC-FID. The result showed that the content of C16:0 and C18:2 was significantly increased, while C18:0 and C18:3 was significantly decreased in OE4 and OE5 compared to WT (Fig. 8I). Together, these results indicate that overexpression of *MSTRG.22563* has a role in increasing seed weight and protein content while decreasing seed oil content and changing the fatty acid composition.

### The change of the expression levels of genes related to lipid metabolism in *MSTRG.22563* overexpression seeds

**Fig. 9:**
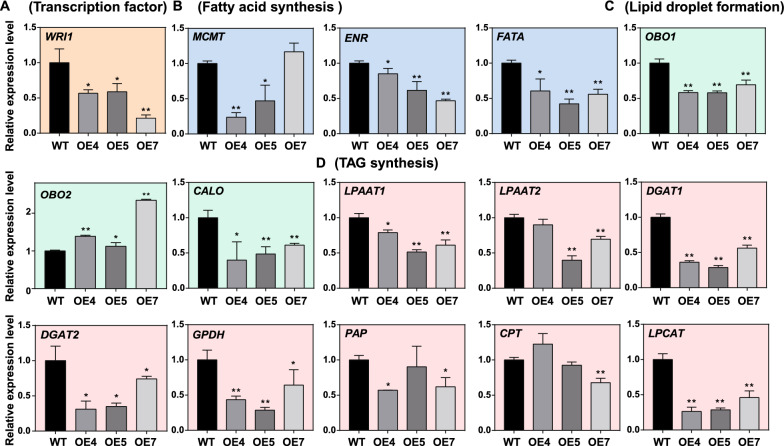
The expression levels of genes involved in lipid synthesis in the 32 DAF seeds of *MSTRG.22563* OE plants. **A** The expression levels of TF. *WRI*, *Wriknkled1*. **B** The expression levels of genes related to fatty acid synthesis. *MCMT*, *ENR*, and *FATA*. *MCMT*, *Malonyltransferase*; *ENR*, *Enoyl-ACP reductase*; *FATA*, *Acyl-ACP thioesterase A*. **C** The expression levels of genes participated in lipid droplet formation. *OBO1*, *Oil body oleosin 1*; *OBO2*, *Oil body oleosin 2*; *CALO*, *Caleosin*. **D** The expression levels of genes involved in TAG assembly. *LPAAT1*, *lysophosphatidic acid acyltransferase 1*; *LPAAT2*, *lysophosphatidic acid acyltransferase 2*; *DGAT1*, *diacylglycerol acyltransferase 1*; *DGAT2*, *diacylglycerol acyltransferase 2*; *GPDH*, *Glycerol-3-phosphate dehydrogenase*; *PAP*, *Phosphatidate phosphatase*; *CPT*, *Choline phosphotransferase*, *LPCAT*, *lysophosphatidylcholine acyltransferase*. Total RNA was extracted from 32 DAF seeds. Relative expression level was calculated by comparison to actin’s expression level. Values are means ± SD (*n* = 3). * and ** denote significance at *P* < 0.05 and *P* < 0.01, respectively, compared with WT based on the student’s *t*-test

To clarify how *MSTRG.22563* regulates the oil accumulation in seeds of *B. napus*, the total RNA was extracted from 32 DAF seeds and the expression of genes was examined by qRT-PCR. The results showed that the expression levels of *WRI1*, *LPAAT1*, *LPAAT2*, *DGAT1*, *DGAT2*, *GPDH*, *PAP*, *LPCAT*, *OBO1*, *CALO*, *MCMT*, *ENR*, and *FATA* were significantly lower in the transgenic seeds than that in WT, except some OE lines had no difference in the expression of *MCMT*, *PAP*, *LPAAT2*, and *CPT* compared to WT (Fig. 9A–D). These results indicate that *MSTRG.22563* suppresses the expression of the key genes in the processes of oil synthesis and thus reduces the oil accumulation in *B. napus* seeds.

### Overexpression of *MSTRG.22563* impacted the content of metabolites in the TCA cycle and respiration

**Fig. 10:**
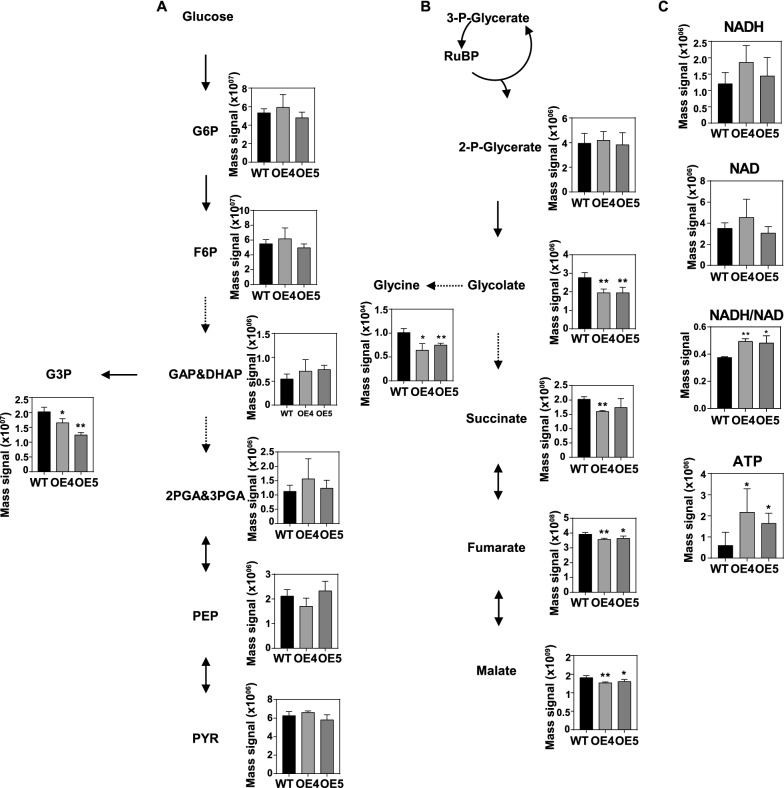
The analysis of metabolites in 32 DAF seeds of *MSTRG.22563* transgenic plants. A The mass signal of metabolites related to glycolysis, including G6P, F6P, GAP&DHAP, 2PGA&3PGA, PEP, PYR, and G3P. Values are means ± SD (*n* = 4–8). **B** The mass signal of metabolites involved in respiration and TCA cycle, including 2-P-glycerate, glycolate, glyoxylate, glycine, malate, succinate, and fumarate. Values are means ± SD (*n* = 4–8). **C** The mass signal of metabolites related to energy metabolism, including ATP, NADH, NAD, and the ratio of NADH/NAD. Values are means ± SD (*n* = 4–8). * and ** denote significance at *P* < 0.05 and *P* < 0.01, respectively, compared with WT based on the Student’s *t*-test. G6P, Glucose-6-phosphate; F6P, Fructose-6-phosphate; DHAP, dihydroxy-acetone-phosphate; GAP, glyceraldehyde-3-phosphate; G3P, glycerol-3-phosphate; 2PGA, 2-Phosphoglycerate; 3PGA, 3-Phosphoglycerate; PEP, Phosphoenolpyruvate; Pyr, pyruvate. TCA, tricarboxylic acid

WRI1 is reported to be related to glycolysis and fatty acid biosynthesis [35, 36]. To analyze the effects of decreased WRI1 on glycolysis process, the total metabolites of 32 DAF seeds were extracted and analyzed using the LC–MS/MS. The results showed that the content of the metabolites in the process of glycolysis such as glucose-6-phosphate (G6P), fructose-6-phosphate (F6P), glyceraldehyde-3-phosphate (GAP), dihydroxy-acetone-phosphate (DHAP), 2-phosphoglycerate (2PGA), 3-phosphoglycerate (3PGA), phosphoenolpyruvate (PEP) and pyruvate (PYR) was not changed in OE4 and OE5 compared to WT (Fig. 10A). While, interestingly, some metabolites such as glycerol-3-phosphate (G3P) (the main substrate of TAG synthesis), glycolate (the product of respiration), succinate, fumarate, and malate (the intermediates of tricarboxylic acid (TCA) cycle) were significantly down-regulated in OE4 and OE5 seeds compared to WT (Fig. 10B). The content of Glycine which is the final metabolic product of glycolate was also decreased in OE seeds (Fig. 10B). However, the metabolites related to energy metabolism such as ATP, and NADH/NAD was significantly increased in OE seeds compared to WT (Fig. 10C). These results imply that *MSTRG.22563* might has a role in regulating the respiration of seeds, resulting in the alternation of metabolites in the TCA process and energy metabolism in seeds.

## Discussion

In the last few years, the rise of research in lncRNAs has attracted a great deal of attention. So far, many lncRNAs have been identified both in plants and animals [21, 37–39]. However, it is still a great challenge to understand the action mechanism of lncRNAs in organisms due to the complex action mechanism on their target [25, 40, 41]. In our study, we tried to find the target of lncRNAs using the co-expression relationship between lncRNA and mRNA. While, the number of highly correlated mRNA was several folds than that of lncRNA. For example, there were 257 mRNAs highly correlated to 42 lncRNAs in cluster 2, and 352 mRNAs highly correlated to 57 lncRNAs in cluster 4 (Fig. 4A, B). In the highly correlated mRNAs, a lot of them were involved in lipid metabolism including *DGAT2*, *KASI*, *KASIII*, *FAX1*, and *LACS8*, and TF like *VAL1* which is reported to regulate lipid metabolism (Fig. 4A, B). It implies that some lncRNAs in cluster 2 and 4 might have roles in regulating lipid metabolism. Previously, some lncRNAs have also been predicted to be related to lipid metabolism in plant seeds by co-expression analysis [33, 42]. Recently, the omics-approaches was reported to be used to assign the functions of lincRNAs [43].

Many studies showed that lncRNA had tissue and species specific [18, 33, 44]. Studies have verified that the major lncRNAs have high sequence conservatism in intra-species and sub-species [45, 46]. Such as, the conservatism of lncRNA is 84.0% between *B. napus* and *B. oleracea*, and 78.8% between *B. napus* and *B. rapa* [33]. A study was reported that there were Brassicaceae-conserved putative miRNA binding motifs in lincRNAs [43], implying the conservatism existed in the lincRNAs of Brassicaceae plants. In this work, the same lncRNA sequence existed in both accessions (Fig. 2A), and the ATAC-seq analysis showed that there were highly consistent open patterns of the lncRNAs in chromatin in four *B. napus* accessions (Fig. 4E, F), indicating that the lncRNAs have highly conservatism in the seeds of different types of *B. napus*. Furthermore, 8094 expressed lncRNAs at three different seed stages in two *B. napus* accessions were screened under the analysis of CNCI, CPC, PFAM, and CPAT. Previous study identified a total of 8905 expressed lncRNAs from 48 RNA-datasets including *B. napus*, *B. oleracea*, and *B. rapa* using the analysis of CPC and PFAM as the screened condition [33]. Comparatively speaking, we obtained more strictly lncRNAs which could present the more realistic lncRNA level in developing seeds. In addition, the number of the lncRNAs in our results was not less too much compared to previous report.

Lots of studies have shown that a large number of genes are involved in the processes of fatty acid and TAG synthesis [47, 48]. Recently, lncRNAs and mRNAs were examined in two lipid accumulation stages in tung tree seeds, and the related chromatin interactions were predicted based on Hi-C data. A total of 68 lncRNAs and 46 genes were included in the regulatory network of lipid biosynthesis [23]. In addition, 1910 lncRNAs were obtained from the full-length transcriptome analysis of five different developmental stages of *Camellia oleifera* seeds [22]. However, it is still unknown whether the lncRNAs play roles in the lipid biosynthesis of plant seeds. Here, we screened two lncRNAs that might be involved in the regulation of lipid metabolism, and the results showed that *MSTRG.86004* promoted oil accumulation while *MSTRG.22563* decreased oil accumulation, and both of them could change the fatty acid composition (Figs. 5, 8). Further researches showed that *MSTRG.86004* could delay the seed development, while *MSTRG.22563* could impact seed respiration to influence the metabolites in the TCA process and energy metabolism (Figs. 7, 10). However, the plant growth was not affected significantly (Additional file 2: Table S24). It implies that the lncRNAs *MSTRG.86004* and *MSTRG.22563* play their roles in the seeds only.

Compared to WT, the *MSTRG.86004* OE seeds had higher expression of genes related to fatty acid biosynthesis during seed development, but lower expression of genes related to lipid metabolism. Surprisingly, the expression of *WRI1* had no difference between OE and WT lines (Fig. 6). However, the seed oil content and weight of *MSTRG.86004* OE plants were significantly higher than that of WT (Fig. 5). Comparison of the same development time seeds showed that the OE seeds were greener, higher water content, and less fatty acid synthesis than WT, but the dry weight was equaled in OE and WT seeds (Fig. 7). These results suggest that the seed development of WT is faster than that of OE at the same time. *LEC1* and *LEC2* are the two marker genes that can indicate seed development [49, 50]. Expectedly, the expression of *LEC1* was much higher in OE seeds than that of WT (Fig. 7D), indicating that the seed development of OE was delayed than the same development time seeds of WT. Additionally, during the seed development period, the *LEC1* usually has a high expression at the early embryonic development, while the time of *WRI1* expression is later than that of *LEC1* [51]. Moreover, *WRI1* can keep a high expression for a longer time during seed development compared to *LEC1* [52]. This phenomenon was also observed in the developing seeds of ZS11 (Additional file 1: Fig. S3). These might be the reason that the expression of *LEC1* was higher but the expression of *WRI1* was no different in *MSTRG.86004* OE seeds compared to WT. Furthermore, the OE mature seeds had higher DAG and TAG but lower phospholipids such as PC, PE, and PS, which is consistent with the content of lipids in the high oil content seed of *B. napus* [34]. A hypothesis is provided that delayed seeds development of *MSTRG.86004* OE plants might need more growth time in silique to finish maturation, then leading to improve the seed weight and seed oil content. In the future, a statistic of the time of the OE seeds from pollination to maturation maybe help us understand the role of *MSTRG.86004* in seeds comprehensively.

The *MSTRG.22563* impacted seed oil accumulation was different from that of *MSTRG.86004*. The qRT-PCR results showed the expression of most genes related to fatty acid biosynthesis and lipid metabolism was significantly decreased in *MSTRG.22563* OE lines compared to WT (Fig. 9). The changes of gene expression pattern in *MSTRG.22563* transgenic seeds are extremely like the changes in *wri1* mutants [53]. The *WRI1* is reported as the most important gene for fatty acid biosynthesis and plays the role of “push” and “pull” in fatty acid metabolism [54]. As expected, the expression of *WRI1* was suppressed seriously in OE lines (Fig. 9A). The mutation of *WRI1* was also reported to suppress the glycolysis in the cytoplasm [53]. However, it was surprising that the metabolites such as G6P, F6P, PEP, and pyruvate in the glycolytic pathway were not changed in OE seeds compared to WT (Fig. 10A). One reason might be related to the metabolites extracted from whole seeds, some metabolites in glycolysis could be suppled from other metabolism process. Interestingly, some metabolites like glycolate, glycine, succinate, fumarate, malate, and G3P were all reduced in OE seeds than that in WT. However, the metabolites related to energy metabolism including ATP and NADH/NAD were increased in OE seeds, compare to WT (Fig. 9). A previous study reported that the glycolate could directly impact succinate metabolism in the TCA process through its metabolic product glyoxylate [55]. Moreover, study also shows that the respiration can impact malate metabolism and reduce the biomass accumulation [56]. These results were consistent with the OE having the less malate content but heavier seed weight (Figs. 8D, 10B). Hence, the reduced respiration might be the main reason for the seed weight was improved in OE.

A previous study reported that GPDHc had a role in modulating the NADH/NAD ratio and impacting the mitochondrial G3P shuttle in plant cells [57]. The NADH/NAD ratio and the content of malate were higher in *atgpdhs* compared to that of WT. Besides, the GPDHc also altered the respiration of Arabidopsis [57]. It seemed that there were countless ties among GPDH activity, respiration, energy metabolism, and TCA cycle [57, 58]. The expression of *GPDH* was lower in *MSTRG.22563* OE seeds than that of WT, which could explain the *MSTRG.22563* OE seeds had low G3P content. However, the content of DHAP in OE was equal to WT seeds. Perhaps, the reasonable explanation was that the respiration impacted the mitochondrial G3P shuttle which induced inhibition of *GPDH* expression in OE seeds. Subsequently, reduced G3P suppressed the whole process of TAG assembly. The signal of the disruption of TAG synthesis feedback to *WRI1*, and then the expression of *WRI1* was suppressed. Therefore, we infer that *MSTRG.22563* might impact the cells’ respiration to reduce the energy loss and then the economized energy feedback to the TCA process to reduce the metabolism in the TCA cycle, at the same time, the reduced glycolate also can act on the TCA process. In the future, it is necessary to distinguish the content of the metabolites in separate subcellular organelles, which will help us understand the detailed role of *MSTRG.22563* in *B. napus* seeds.

## Conclusions

Seed oil content is one of the most important breeding goals in *B. napus*, which is the third oil crop in the world. In the past few decades, the functions of many genes and TFs were well studied in seed oil accumulation. However, the research on the transcriptional regulation in seed oil biosynthesis is limited. LncRNAs were reported to regulate many biological processes, such as plant growth, development, and abiotic and biotic stresses tolerance. Recently, lncRNAs were also predicted to take part in regulating seed oil accumulation. While, there was no experimental verification to verify the relationship between lncRNAs and seed oil content. In this study, we totally identified 8094 expressed lncRNAs in three developing seeds of two *B. napus* accessions, and the experimental data showed that lncRNAs *MSTRG.22563* and *MSTRG.86004* could impact seed oil content of *B. napus*. Together, our results of two lncRNAs had roles in regulating oil accumulation and fatty acid composition of *B. napus* seeds would provide a new insight to understand the regulation mechanism in TAG metabolism.

## Methods

### Plant growth condition, seeds collection, RNA sequencing and trait measurements

Developing seeds at 10, 24, and 34 days after flowering (DAF) of *B. napus* accessions ZS11 (high oil content) and WH5557 (low oil content) were collected from the field under natural conditions (three replicates for each line). The detail information of the oil content and fatty acid composition of ZS11 and WH5557 seeds were described previously [34]. Total RNA was extracted from the seeds using TRIzol reagent (Invitrogen, USA) and treated with DNase (Promega, Madison, WI) according to the manufacturer’s instructions. Total RNA samples were then tested by (1) 1% agarose electrophoresis for degradation and impurities, (2) Keo K5500 spectrophotometer for sample purity (Keo, Beijing), and (3) Agilent 2100 RNA Nano 6000 Assay Kit (Agilent Technologies, CA, USA) for RNA sample integrity and concentration. Qualified RNA was sent to the instrument to do the RNA sequencing (http://www.genome.cn/). The steps of library construction were followed by the flow chart in Additional file 1: Fig. S1. Finally, the constructed library was sequenced by Illumina sequencing.

The seeds of transgenic plants and WT were sown in the field in Huazhong Agricultural University to set a plot experiment, and the field is approved for planting transgenic plant by local government. Each material was planted three lines (10 plants per line) with the same spacing from one plant to the other. Total RNA and metabolites were extracted from 32 DAF seeds of transgenic plants, while the comparison of seed development situation used 40 DAF seeds from *MSTRG.86004* transgenic plant. Mature seeds were used for analysis of the seed oil content, protein, TAG and phospholipids. Five to ten plants were harvested from each material, and the agronomic traits such as plant height, inflorescence length, and silique length of each line were averaged and used of ANOVA analysis and Duncan’s multiple range test.

### Identification of lncRNAs

The adaptor-polluted reads, low-quality reads, splice contamination, etc. were filtered out from Raw Data to produce clean data as previously described [59]. The genome of *B. napus* Darmor-*bzh* was used as a reference [60]. The clean data were mapped to the reference genome using HISAT2 (http://ccb.jhu.edu/software/hisat2/index.shtml). A software StringTie was employed to rapidly assemble transcripts as previously described [61]. The transcripts were then screened according to four basic criteria: (1) transcript length greater than or equal to 200 bp and exon number greater than or equal to 2, (2) the coverage per transcript reads was calculated, and the number of transcripts less than 5 in all samples were deleted, (3) known mRNAs and other non-coding RNAs (rRNA, tRNA, sRNA) were screened out using gffcompare compared to the annotation file, and (4) screened the potential lincRNAs, intronic lncRNAs, and antisense lncRNAs based on the class_code information (“u”, “i”, “x”) in the comparison results. The sequences from the initial screening in the step above were combined with four coding potential analysis software including Coding–Non-Coding Index (CNCI), Coding Potential Calculator (CPC), Coding Potential Assessment Tool (CPAT), and pfamscan to analyze whether the sequences have coding potential. The transcripts judged as non-coding RNAs by the four analysis methods above were then made for the Venn analysis. The RNAs that existed in the four analysis methods were the lncRNAs.

### Quantitation of lncRNA and mRNA, and the analysis of differentially expressed genes

Read count for each lncRNA or mRNA was counted by FPKM (Fragments Per Kilobase Million Mapped Reads). Original read count was used for the differential expression genes analysis to compare the expression of lncRNA or mRNA among three different developing seeds of the same accession, or the same seed development stage of ZS11 and WH5557. LncRNAs or mRNAs with |log_2_foldchange|≥ 1 and *q* < 0.05 were identified as significantly differentially expressed genes.

### t-SNE and GO analysis

R (t-SNE) packages were employed to do the reduced-dimensional clustering analysis of gene expressions of samples from different varieties at different times [62]. All *B. napus* genes were used to compare with all *A. thaliana* proteins using BLASTP [63]. The selection of the most essential proteins in *A. thaliana* (with a probability threshold of 10^–5^) was enriched with the *A. thaliana* genome. The enrichment analysis was then performed using Fisher’s exact test and OR and *P* values were calculated.

### Circos analysis and lncRNA–mRNA co-expression

Gene expression and genome-wide number distribution are visualized using Circos v.0.69–6 [64]. We used the Mfuzz package with Default Parameters in R to perform cluster analysis using the FPKM values of expressed genes and list the most significant GO terms in each cluster [65].

### Quantitative real-time PCR (qRT-PCR)

Total RNA was extracted from 32 DAF *B. napus* seeds using a qRT-PCR kit (Promega, Carlsbad, CA, USA). First-strand cDNA was synthesized using EasyScript RT Kit (AE311-03). qPCR was performed using the BIO-RAD CFX96 qPCR detection system (BIO-RAD, HTTP: // www.bio-rad.com), and SYBR green was used to monitor dsDNA synthesis. Primers used for qPCR are listed in Additional file 2: Table 23. qPCR conditions were followed the previous description [34].

### The vectors construction and plant transformation

The sequence of *MSTRG.22563* and *MSTRG.86004* was cloned by PCR using the cDNA of 34 DAF seeds of WH5557. The sequence of *MSTRG.22563* and *MSTRG.86004* were then linked to the pMDC83 binary vector, respectively. The constructed vector was transferred into *Agrobacterium tumefaciens* GV3101, which was used to infect the hypocotyl of *B. napus* according to the protocol as described previously [66]. Transgenic plants were subsequently tested with specific primers and explanted into the field. The primers used above are shown in Additional file 2: Table S23.

### Lipid extraction and LC–MS analysis

Lipids were extracted from mature seeds as described previously [67]. Finally, the extracted lipid was dried with nitrogen and transferred to a 2 ml sample bottle. The dry lipid extract was diluted to 2 mg/ml based on dry seed weight and was injected into LC–MS/MS (AB sciex 6500 plus) for lipid analysis. The parameters and running conditions of the instrument were described previously [67].

### Metabolite extraction and LC–MS analysis

Metabolite extraction and analysis were followed the previously described method with a little modified [68]. Added 3 mg of lyophilized 32 DAF seeds to 3 mL methanol-chloroform (7:3, *v/v*) and incubated at − 20 °C for 2 h. Added 2.4 mL ddH_2_O to the tube and mixed, centrifuged at 500 g for 5 min, then transferred the upper phase to a new glass tube. Repeated the step above. Dried the aqueous phase with nitrogen, 200 μL ddH_2_O was added to dissolved again. The solution was diluted tenfold for metabolite analysis by LC–MS/MS.

## Analysis of seed oil content, protein content and fatty acid composition

The seed oil content and protein content of mature seeds was determined using a near-infrared instrument. The fatty acid composition of mature and developing seeds was measured by a gas chromatography-flame ionization detector (GC-FID) [69].

## Supplementary Information

**Additional file 1: Figure S1.** Library construction flow chart. **Figure S2.** The flow chart of transcripts’ analysis. **Figure S3.** The analysis of top 10 GO terms in different clusters. **Figure S4.** The content of lysoPC and lysoPE in the mature seeds of *MSTRG.86004*. Values are means ± SD (*n* = 8–12). * and ** denote significance at *P *< 0.05 and *P *< 0.01, respectively, compared with WT based on the student’s *t*-test*. Figure S5.* The gene expression levels in the whole seed development stages of ZS11 and 32 DAF seeds of *MSTRG.86004 *OE plants. **A**, The transcripts’ levels of. *WRI1*, *LEC1*, and *LEC2 *in the whole seed development stages of ZS11. Data from. http://yanglab.hzau.edu.cn/BnTIR. **B**, The expression of *LEC2 *in 32 DAF seeds of. *MSTRG.86004 *OE plants. Values are means ± SD (*n* = 3). *WRI1*, *wrinkled 1*; *LEC1*, *leafy cotyledon 1*; *LEC2*, *leafy cotyledon 2*. **Figure S6.** The dry weight and water content in 40 DAF seeds of *MSTRG.22563 *overexpression plants. Values are means ± SD (*n *= 6-8). **Figure S7.** The content of lysoPC and lysoPE in the mature seeds of *MSTRG.22563. *Values are means ± SD (*n* = 8–12). * and ** denote significance at *P *< 0.05 and *P *< 0.01, respectively, compared with WT based on the student’s *t-test*.

**Additional file 2: Table S1.** Transcripts obtained by CNCI screening. **Table S2.** Transcripts obtained by CPC screening. **Table S3.** Transcripts obtained by CPAT screening. **Table S4.** Transcripts obtained by PFAM screening. **Table S5.** The lncRNAs obtained from the intersection of the four lncRNA analysis methods. **Table S6.** LncRNAs and their type on chromosome after screening by variable splicing. **Table S7.** The expression level of Mrna. **Table S8.** The expression level of lncRNA. **Table S9.** GO details for cluster 1. **Table S10.** GO details for cluster 2. **Table S11.** GO details for cluster 3. **Table S12.** GO details for cluster 4. **Table S13.** GO details for cluster 5. **Table S14.** GO details for cluster 6. **Table S15.** GO details for cluster 7. **Table S16.** GO details for cluster 8. **Table S17.** GO details for cluster 9. **Table S18.** GO details for cluster 10. **Table S19.** GO details for cluster 11. **Table S20.** GO details for cluster 12. **Table S21.** The specifics of co-expression within cluster 2. **Table S22.** The specifics of co-expression within cluster 4. **Table 23.** The primers used in this study.

## Data Availability

All the RNA-seq raw sequencing data generated in this study are available in the GenBank Nucleotide Sequence Databases (https://www.ncbi.nlm.nih.gov/genbank/) with Bioproject IDs PRJNA853177.
