# Supplementary material for: Regulation of seed oil accumulation by lncRNAs in Brassica napus
Source: Biotechnol Biofuels Bioprod. 2023 Feb 10;16:22. doi: 10.1186/s13068-022-02256-1 (PMC9921586; doi:10.1186/s13068-022-02256-1)
Supplement: Supplementary file 1 — Additional file 1: Figure S1. Library construction flow chart. Figure S2. The flow chart of transcripts’ analysis. Figure S3. The analysis of top 10 GO terms in different clusters. Figure S4. The content of lysoPC and lysoPE in the mature seeds of MSTRG.86004. Values are means ± SD (n = 8–12). * and ** denote significance at P < 0.05 and P < 0.01, respectively, compared with WT based on the student’s t-test. Figure S5. The gene expression levels in the whole seed development stages of ZS11 and 32 DAF seeds of MSTRG.86004 OE plants. A, The transcripts’ levels of. WRI1, LEC1, and LEC2 in the whole seed development stages of ZS11. Data from. http://yanglab.hzau.edu.cn/BnTIR. B, The expression of LEC2 in 32 DAF seeds of. MSTRG.86004 OE plants. Values are means ± SD (n = 3). WRI1, wrinkled 1; LEC1, leafy cotyledon 1; LEC2, leafy cotyledon 2. Figure S6. The dry weight and water content in 40 DAF seeds of MSTRG.22563 overexpression plants. Values are means ± SD (n = 6-8). Figure S7. The content of lysoPC and lysoPE in the mature seeds of MSTRG.22563. Values are means ± SD (n = 8–12). * and ** denote significance at P < 0.05 and P < 0.01, respectively, compared with WT based on the student’s t-test. [file 13068_2022_2256_MOESM1_ESM.pdf]

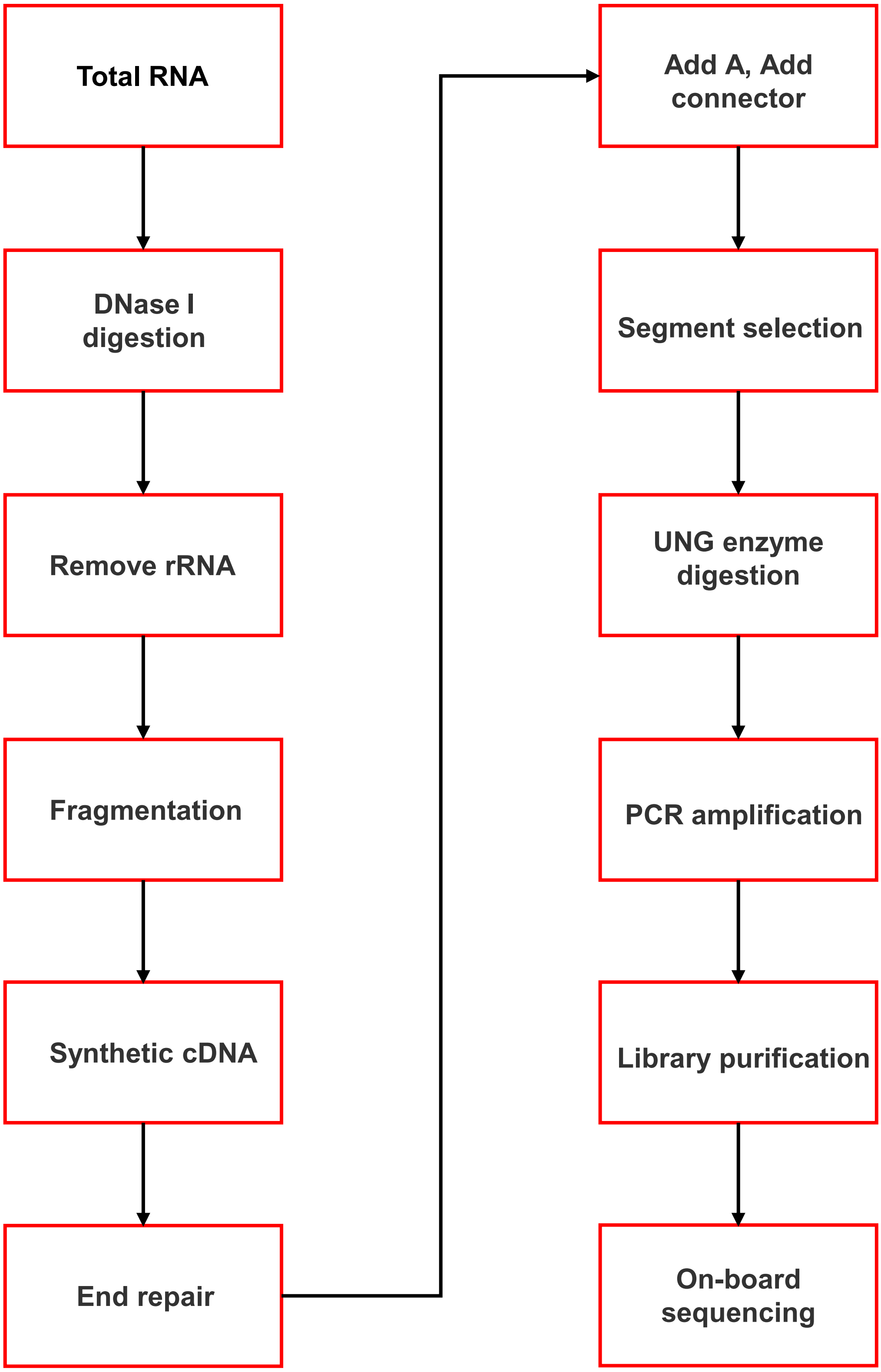

Figure S1. Library construction flow chart.

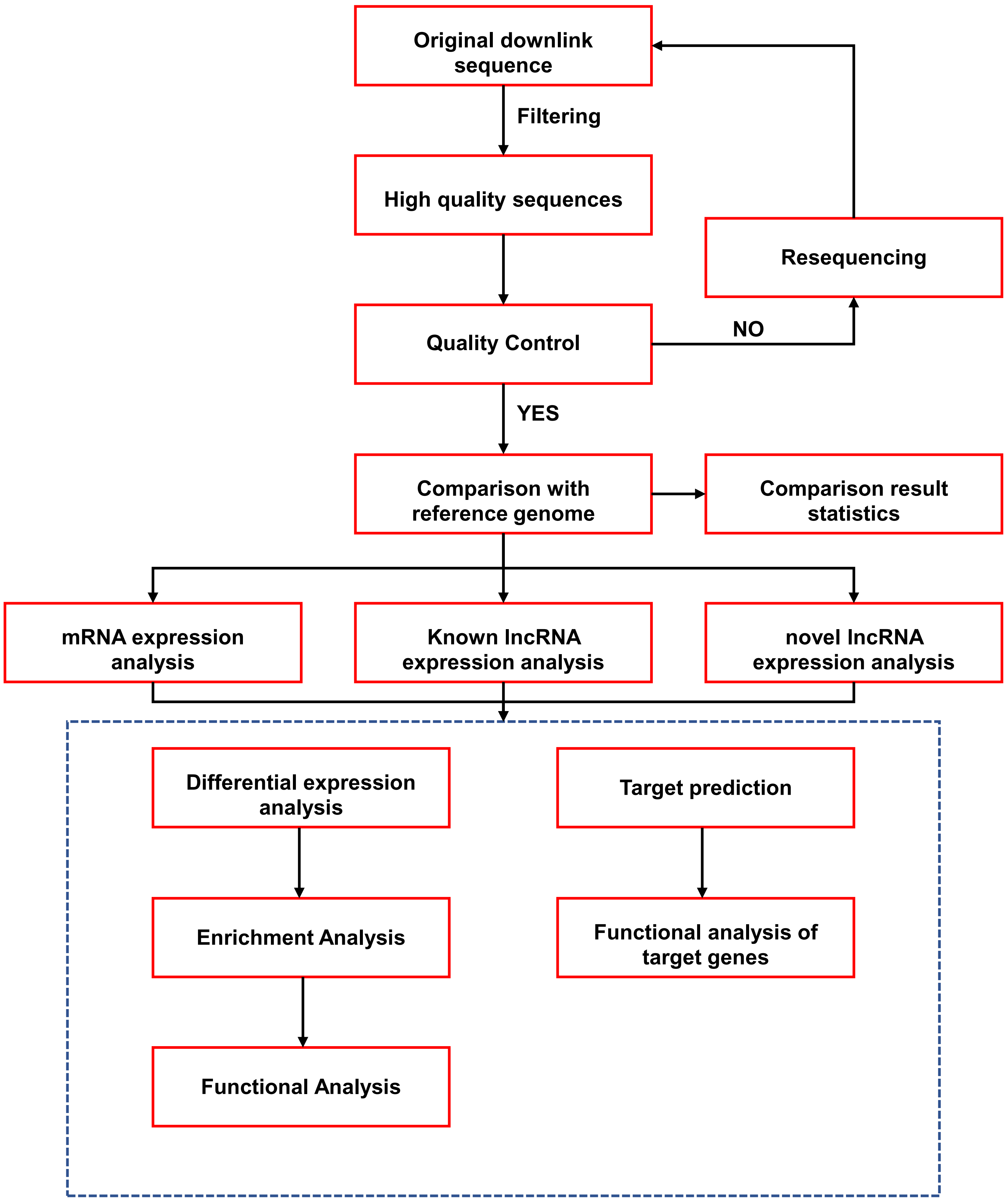

Figure S2. The flow chart of transcripts' analysis.

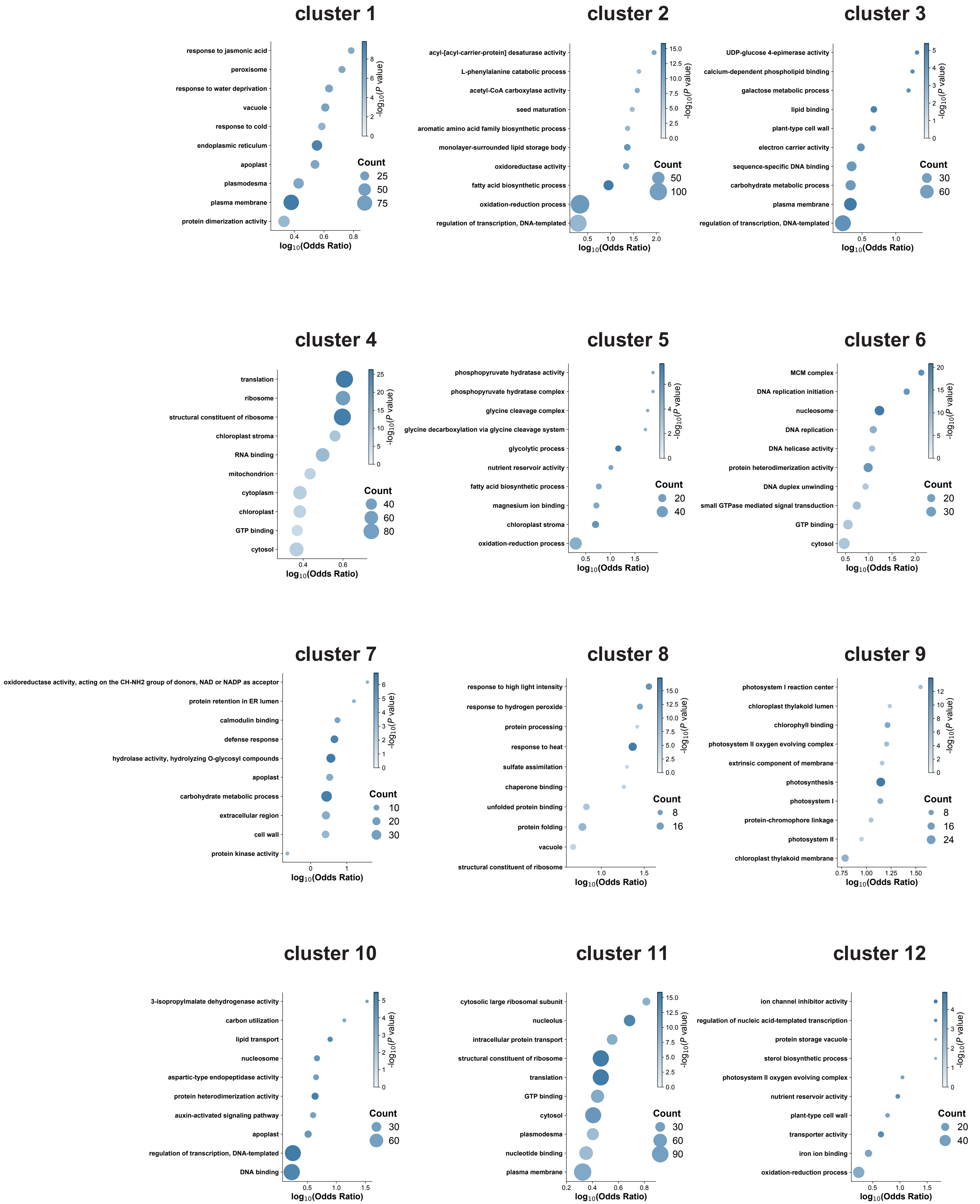

Figure S3. The analysis of top 10 GO terms in different clusters.

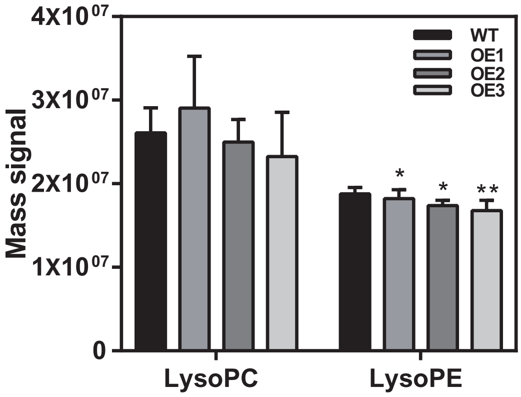

Figure S4. The content of lysoPC and lysoPE in the mature seeds of *MSTRG.86004*. Values are means ± SD (n = 8-12). * and ** denote significance at $P < 0.05$ and $P < 0.01$, respectively, compared with WT based on the student's *t-test*.

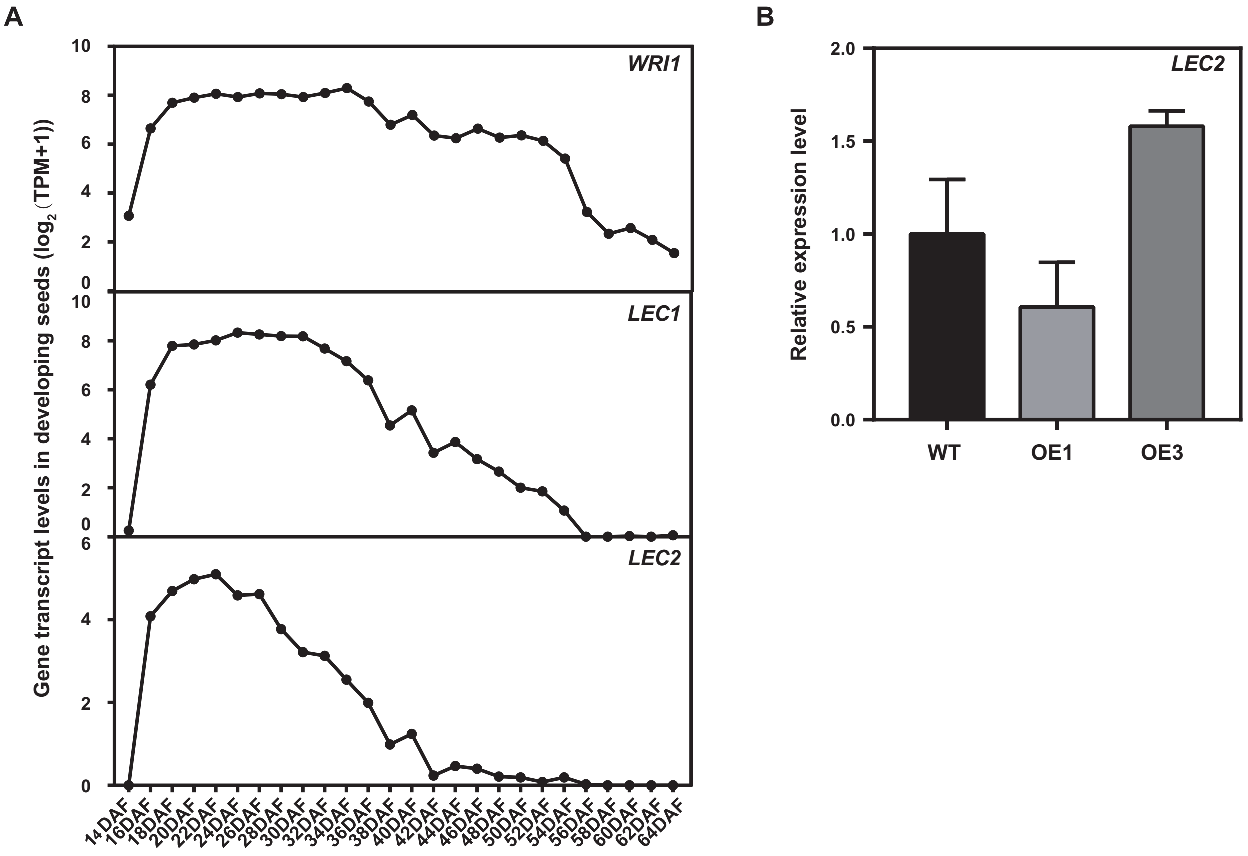

Figure S5. The gene expression levels in the whole seed development stages of ZS11 and 32 DAF seeds of *MSTRG.86004* OE plants. A, The transcripts' levels of *WRI1*, *LEC1,* and *LEC2* in the whole seed development stages of ZS11. Data from http://yanglab.hzau.edu.cn/BnTIR. B, The expression of *LEC2* in 32 DAF seeds of *MSTRG.86004* OE plants. Values are means ± SD (n = 3). *WRI1*, *wrinkled 1*; *LEC1*, *leafy cotyledon 1*; *LEC2*, *leafy cotyledon 2*.

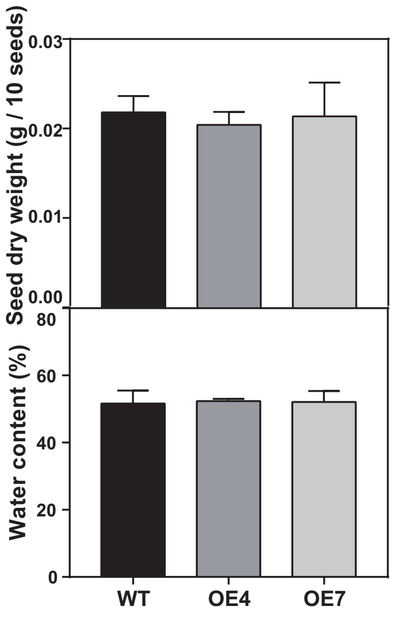

Figure S6. The dry weight and water content in 40 DAF seeds of *MSTRG.22563* overexpression plants. Values are means ± SD (*n* = 6-8).

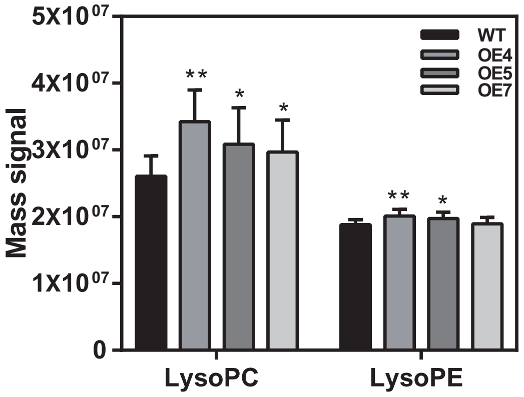

Figure S7. The content of lysoPC and lysoPE in the mature seeds of *MSTRG.22563*. Values are means ± SD (n = 8-12). * and ** denote significance at $P < 0.05$ and $P < 0.01$, respectively, compared with WT based on the student's *t-test*.
